# Synthesis and Application of Silver Nanoparticles (Ag NPs) for the Prevention of Infection in Healthcare Workers

**DOI:** 10.3390/ijms20153620

**Published:** 2019-07-24

**Authors:** Shingo Nakamura, Masahiro Sato, Yoko Sato, Naoko Ando, Tomohiro Takayama, Masanori Fujita, Masayuki Ishihara

**Affiliations:** 1 Division of Biomedical Engineering, National Defense Medical College Research Institute, Saitama 359-8513, Japan; 2 Section of Gene Expression Regulation, Frontier Science Research Center, Kagoshima University, Kagoshima 890-8544, Japan; 3 Department of Oral and Maxillofacial Surgery, National Defense Medical College Hospital, Saitama 359-8513, Japan; 4 Division of Environmental Medicine, National Defense Medical College Research Institute, Saitama 359-8513, Japan

**Keywords:** antiviral property, healthcare workers (HCWs), medical application, microbicidal property, silver nanoparticles (Ag NPs), cytotoxicity

## Abstract

Silver is easily available and is known to have microbicidal effect; moreover, it does not impose any adverse effects on the human body. The microbicidal effect is mainly due to silver ions, which have a wide antibacterial spectrum. Furthermore, the development of multidrug-resistant bacteria, as in the case of antibiotics, is less likely. Silver ions bind to halide ions, such as chloride, and precipitate; therefore, when used directly, their microbicidal activity is shortened. To overcome this issue, silver nanoparticles (Ag NPs) have been recently synthesized and frequently used as microbicidal agents that release silver ions from particle surface. Depending on the specific surface area of the nanoparticles, silver ions are released with high efficiency. In addition to their bactericidal activity, small Ag NPs (<10 nm in diameter) affect viruses although the microbicidal effect of silver mass is weak. Because of their characteristics, Ag NPs are useful countermeasures against infectious diseases, which constitute a major issue in the medical field. Thus, medical tools coated with Ag NPs are being developed. This review outlines the synthesis and utilization of Ag NPs in the medical field, focusing on environment-friendly synthesis and the suppression of infections in healthcare workers (HCWs).

## 1. Introduction

Silver is widely used in industrial applications because of its metallic properties, such as conductivity, and in the medical field due to its antimicrobial effect [1]. Silver shows antibacterial activity against various organisms, and this effect is observed even at low concentrations [2]. Berger et al. reported that the growth of *Escherichia coli* (*E. coli*), *Staphylococcus*, *Providencia*, *Serratia*, and *Pseudomonas aeruginosa* is inhibited by the presence of ~1 μg/mL silver ions [1]. Ip et al. reported that several wound-coating materials (wound dressings) that contain silver exert an antibacterial effect against methicillin-resistant *Staphylococcus aureus* (MRSA) [2]. The antibacterial activity of silver is mainly attributed to silver ions, which are released from a silver-containing substance and interact with the thiol groups of enzymes and proteins that support bacterial life, thus affecting cell respiration and killing the cells [3]. When halide ions, such as chloride, are present in the environment, silver ions bind to them and precipitate, losing their water solubility and antibacterial activity. Therefore, the antibacterial activity of free silver ions is very short when they are used alone [4]. To overcome this issue, silver nanoparticles (Ag NPs) [5], silver-containing fine glass particles [6], and Ag NPs/chitin or chitosan complexes [7,8,9] have recently been developed. From these compounds, silver ions are gradually released, thereby causing antimicrobial activity.

For a metallic particle to be considered as “nano”, its size must be within 1–100 nm [10]. Metallic nanoparticles exhibit specific properties, such as surface plasmon resonance [11]. Such particles have been used for glass decoration because of their specific vivid color, e.g., red from gold nanoparticles and yellow from Ag NPs. In addition, nanoparticles have a large specific surface area with a small amount of metal mass [5]. Metal nanoparticles are commonly synthesized via the reduction of metal salts in a solution [12] or the formation of metal atom aggregates by the heating or vaporization of a metal in inert gas or vacuum [13]. Size-controlled Ag NPs are also synthesized using new environment-friendly synthesis techniques [14]. The surface of Ag NPs in an aqueous environment is oxidized in the presence of oxygen and protons, and silver ions are released as the surface dissolves. Thus, the effective silver ion concentration is maintained in the solution, and the antimicrobial effect will last for a long time [5].

Various applications have been found in the medical field for Ag NPs; for example, they can be used for biosensors, drug delivery systems, and medical devices [15,16,17]. Because of their wide antibacterial and antiviral spectrum, there are particularly high expectations for the suppression of multidrug-resistant bacteria. For example, Ag NPs are combined with a cationic polymer to produce a bactericidal material. Ag NPs have good applicability and are easily processed because of their low melting point [18]. This review describes representative methods used for synthesizing Ag NPs, focusing on environment-friendly synthesis, their effect on microorganisms and viruses, and their application to medical devices, particularly the suppression of infections in both patients and healthcare workers (HCWs).

## 2. Synthesis of Ag NPs

**Figure 1:**
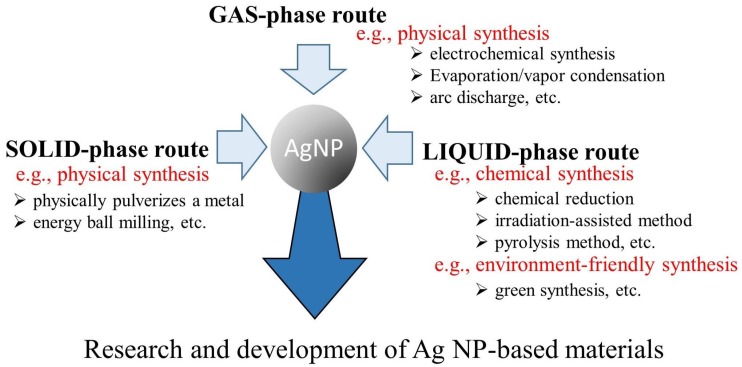
Various methods for silver nanoparticle (Ag NP) synthesis. Ag NPs have been synthesized using various methods that can be classified as solid, liquid, or gas-phase routes.

Ag NPs have been synthesized using various methods, which can be classified as gas or aerosol, solid, and liquid-phase routes (Figure 1). Both chemical and physical synthesis methods for Ag NPs are well known [19]. In recent years, green synthetic pathways have also been proposed [20]. These green processes reduce the generation of harmful byproducts that damage the environment. They also allow for an efficient resource-saving synthesis.

### 2.1. Environment-Friendly Synthesis Methods

The green synthesis method based on green chemistry programs is known as the representative environment-friendly synthesis method. To avoid the use or discharge of hazardous chemical substances to a considerable extent during the synthesis of chemical compounds, green chemistry programs were proposed by the Environmental Protection Agency of the United States (EPA) in 1990. Then, in 1998, Anastas and Warner published the “Twelve Principles of Green Chemistry”, summarizing the concept of green chemistry [21]. Since then, policies concerning the handling of environment-friendly chemical substances have been announced worldwide. Moreover, green sustainable chemistry advocates for resource savings by recycling, which is not necessarily covered by green chemistry. This consideration has also become widespread in the materials science field, and reports on the green synthesis of Ag NPs have increased. The components of these materials such as nicotinamide adenine dinucleotide (NAD) are capable of reducing Ag salts (silver ions) into Ag NPs. Nicotinamide adenine dinucleotide-dependent reductase can produce Ag NPs by enzymatic reduction; however, the enzymatic reduction rate is often slow [22]. Some biological materials using green synthesis methods using bacteria [23,24,25,26,27,28,29], fungi [30,31,32,33,34,35], and plant [36,37,38,39,40,41,42] are shown in Table 1.

Among the available green methods of synthesis for Ag NPs, utilization of plant extracts is a rather simple and easy process to produce nanoparticles at large scale relative to bacteria and/or fungi mediated synthesis [43]. Several studies have discussed the synthetic conditions, such as pH and reaction temperature, that promote the synthesis of Ag NPs [16]. With regard to pH, polysaccharides and proteins related to the reaction are denatured under strongly acidic conditions; thus, neutral or slightly alkaline conditions are desirable [44,45]. Regarding the temperature of the reaction system, the amount of reactant consumed remarkably increases at high temperatures, yielding nanoparticles [46].

**Figure 2:**
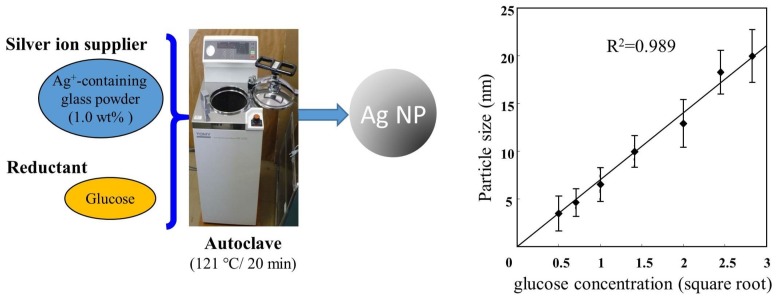
Environment-friendly method for Ag NP synthesis with the diameters control by glucose concentration. We have reported that environment-friendly processes were used to produce small Ag NPs (<10 nm) within a narrow size distribution. The diameters of generated Ag NPs were easily controlled by glucose concentrations [14].

The diameter of the Ag NPs is known to influence the microbicidal effect [15,17]. The bactericidal activity is stronger when the particle size is smaller [47]. Therefore, an adequate synthesis method is required to generate small-size particles (<10 nm) with small dispersions. Ishihara et al. reported that when synthesizing Ag NPs by common autoclaving using commercially available glass powders containing silver nitrate as a silver ion supplier and glucose as a reducing agent, the particle size can be controlled easily depending on the glucose concentration. Ag NPs of 5 ± 1 nm can be efficiently synthesized by the method (Figure 2) [14,48]. Moreover, no harmful material was generated by the synthesis method.

### 2.2. Chemical Synthesis Methods 

Some chemical methods for the synthesis of Ag NPs include chemical reduction [12,49,50,51,52,53,54], electrochemical synthesis [55,56,57], the irradiation-assisted method [58,59,60,61], and the pyrolysis method [62,63], as summarized in Table 2. 

Among them, chemical reduction is well known and requires two main components: a reducing agent and a silver source for the reaction. Various reagents are used for reducing agents [12,50,51]. Of these, borohydride is the most widely used because of its extremely strong and rapid reducing action [12,64,65,66,67]. In addition, Ag NPs synthesized using a co-reduction approach (e.g., sodium borohydride/trisodium citrate [52], hydrazine hydrate/sodium citrate [53], and borohydride/citrate [54]) were reported. Agnihotri et al. reported that Ag NPs were synthesized employing sodium borohydride as a primary reductant and trisodium citrate both as secondary reductant as well as protective agent [52]. In this method, nucleation and growth kinetics during the synthesis process were precisely controlled and Ag NPs of average size 5, 7, 10, 15, 20, 30, 50, 63, 85, and 100 nm were synthesized with good yield and monodispersity. As described, a protective agent is often added to stabilize the produced nanoparticles in a dispersed state. The crystal structure can also be controlled using this protective agent: Rhomboid structures and nanosheets can be produced in addition to the general spherical shape. Silver nitrate, which is chemically stable, easily available, and inexpensive compared to other silver salts [68], can supply silver ions to synthesis systems and is frequently used as the silver source.

Instead of reducing agents, a silver source is also reduced by electrochemical reaction, irradiation-assisted reaction, and pyrolysis reaction. For example, an electrochemical method, which can be used to produce certain transition metal colloids in the nanometer region, was demonstrated for the first time [55]. Ag NPs of ≤ 20 nm can be synthesized using an electrochemical method [55,56]. Using this method, Zhang et al. reported new modification method of ultrathin zeolite film of about 400 nm in thickness; the films are promising candidates for use in membrane applications [56]. Recently, Ag NPs were prepared by an electrochemical method using only polyethylene glycol as a stabilizer and without any other reactant [57]. Interestingly, using irradiation-assisted method, Ag NPs can be extended easily to synthesize relatively monodisperse triangular silver nanocrystals with desired edge lengths in the 30–120 nm range [58] and cubic crystal with 2–8 nm lengths [59]. In addition, irradiation-assisted method contributive to shortening of the synthesis time. According to Manikprabhu and Lingappa, Ag NPs were synthesized rapidly in just 90 s from 20 min by a microwave irradiation method, using pigment as a reducing agent [60]. Zhou et al. reported that nanosilver/gelatin/chitosan hydrogels were prepared by radiation crosslinking and reduction simultaneously, resulting in a stable and homogeneous distribution of Ag NPs in the matrix [61]. To synthesize fine powders by aerosol decomposition, ultrasonic spray pyrolysis has been used; however, a particle size of less than 20 nm has not been reported [62]. Pingali et al. reported that one-step spray pyrolysis of ultrasonically-atomized dilute solutions of metal solutes represents a potentially viable means of generating relatively monodisperse particles, with the capability of obtaining a mean particle size less than 20 nm [62]. Sotiriou et al. also reported that Ag NPs (less than 20 nm) were made and immobilized on nanostructured SiO2 [63].

### 2.3. Physical Synthesis Methods

Briefly, nanoparticles are synthesized using a physical method that physically pulverizes a metal [22,69,70]. Compared to chemical methods, thin films and the uniformity of nanoparticles distribution can be prepared with the absence of solvent contamination; however, a stable high energy over a long time should be supplied in physical methods to produce a high yield of Ag NPs of uniform size, and require large space for equipment. Evaporation/vapor condensation [22,71,72], arc discharge [73], and energy ball milling [74] are some of the commonly used methods for synthesizing nanoparticles. Tien et al. reported the synthesis of 20–30 nm diameter of Ag NPs via arc discharge with no added surfactants [73]. The fabrication consumes silver rods at a rate of 100 mg/min, yielding metallic silver nanoparticle and ionic silver with concentrations of approximately 11 ppm and 19 ppm, respectively. Nakamura et al. developed a simple and rapid synthesis technique (20-min irradiation), via laser irradiation of an aqueous solution of inorganic ions for nanoparticles synthesis [75]. As a result, antibacterial calcium phosphate sub-microspheres containing Ag NPs expected to be useful in dental healthcare and infection control were produced with one-pot fabrication.

## 3. Microbicidal Properties of Ag NPs

The mechanisms of the microbicidal activity of Ag NPs have only recently been understood. Sondi and Salopek-Sondi were the first to report the bactericidal ability of Ag NPs against Gram-negative bacteria, using *E. coli.* They revealed that the nanoparticles accumulate in “pits” that are formed in the cell wall; then, the release of free radicals from the Ag NPs damages the cell and annihilates the bacteria [5]. Furthermore, the redox reaction, in which the silver ions are released from Ag NPs, is also mentioned as one of the factors that damages bacteria [76]. Small Ag NPs (less than 10 nm) releases silver ions from its surface that indicate much higher antibacterial activity than from direct bacterial contact with that surface [77]. Matteis et al. reporeted that the death following the application of Ag NPs is dose-dependent [51]. Silver ions are known to specifically react with the thiol group of cysteine; thus, the metabolic enzymes inside the bacteria are considered to be inhibited. This may be explained either by the formation of new bonds between the silver ions and cysteine residues in the peptide, or by the fact that the ions replace other metal ions already bound to the cysteine, killing the bacteria. The relation between the size of the Ag NPs and the antibacterial effect has also been elucidated, indicating that a smaller diameter results in a higher bactericidal activity [78]. This is likely due to the structure and size of the “pits” on the cell wall, which varies depending on the types of bacteria; smaller Ag NPs can access the “pits” more efficiently than larger Ag NPs [79]. According to Gurunathan et al., in experiments using Ag NPs of 5 nm on average, nanoparticles were more effective than ampicillin or vancomycin against some bacteria [80]. This suggests that Ag NPs are useful against infectious diseases. Ag NPs [81] have a high antibacterial activity against *E. coli* O157: H7 [82], which has a very strong food poisoning effect, and this bactericidal activity is exerted against *Streptococcus pyogenes*, *Salmonella enterica*, *Staphylococcus aureus*, and *Enterococcus faecalis* [83]. Although silver ions show a strong microbicidal activity against prokaryotic cells, particularly Gram-negative bacteria, their activity against Gram-positive bacteria is considered weak. This is explained by the thick cell wall of Gram-positive bacteria. Further, the peptidoglycan in their wall has a significant influence. The affinity between silver ions and the peptidoglycan is very high, and silver ions are presumably trapped in the cell wall and do not reach inside the cell membrane [84]. Furthermore, high temperatures, as well as the presence of chlorine, thiol groups, and oxygen-carrying proteins, strongly influence the presence of silver ions [5]. Thus, the environment in which Ag NPs are used is also an important factor influencing the microbicidal activity of Ag NPs.

## 4. Antiviral Properties of Ag NPs

Studies on the antiviral action of Ag NPs are far behind those targeting microbicidal properties, and the mechanism of antiviral action is still not well understood. A viral infection is established when the nucleic acids of the virus are introduced into the host cell and then replicated. Ag NPs possibly act on the surface of the virus and physically inhibit the contact with host cells [82,83,85]. Previous studies have demonstrated that the size of the Ag NPs is essential for the manifestation of antiviral effects, similar to the observations in bacteria. According to Speshoc et al., ≤25 nm Ag NPs are effective against arenavirus, inhibiting its replication process [86]. Gaikwad et al. indicate that Ag NPs of 7–20 nm have antiviral effects against herpes simplex virus (HSV) types 1/2 and human parainfluenza virus type-3 [87]. Furthermore, since the antiviral effect decreased with increasing particle diameter, the nanoparticles should be as small as possible. Baram-Pinto et al. reported that Ag NPs inhibit the contact of HSV-1 with the cell surface and prevent infection [88]. Ag NPs are effective against cells already infected with human immunodeficiency virus (HIV) [89]. Ag NPs adhere to the envelope of the HIV virus to prevent cell infection [90]. Mori et al. reported that Ag NPs of ≤ 10 nm were effective against the influenza virus [9,14]. Rogers et al. reported that Ag NPs of approximately 10 nm inhibit Monkeypox virus (MPV), an orthopoxvirus similar to variola virus, infection in vitro [91]. Interestingly, they also reported that larger Ag NPs (25 nm, 55 nm, and 80 nm) promoted an increase in the mean number of MPV plaque-forming unit (PFU)/well when compared to controls. A potential explanation for this may be due to nanoparticle agglomeration, the nanoparticle agglomeration may potentiate or facilitate virus particle interaction or internalization within host cells, leading to an increase in the number of observed PFU. Therefore, particle size of Ag NPs seems very important for antiviral properties of Ag NPs. More studies on the antiviral activity of Ag NPs will possibly be reported in the future.

## 5. Toxicity of Ag NPs in Humans 

Investigations on the toxicity of Ag NPs to the human body have only recently been reported. Recent in vitro studies demonstrate the cytotoxicity of Ag NPs against HaCaT (Human keratinocyte cell line) cells, toxicity data in terms of cell viability revealed a dose-dependent safe profile for low concentrations (<10 μM), whereas higher concentrations were associated with a high rate of cell mortality [92]. The evaluation of cytotoxicity of Ag NPs has been also carried out in other human cellular models such as lung fibroblasts [93], glioblastoma cells [93], and mesenchymal stem cells [94]. Oxidative stress and severe lipid peroxidation have been observed, and they certainly damage proteins [95]. The proposed mechanism by which Ag NPs lead to cytotoxicity has been considered to at least partially be through the induction of reactive oxygen species (ROS) [96]. Overproduction of ROS causes impairments in DNA, lipids, and protein, eventually leading to cell death and progressive aging of an organism [97]. In addition, the cells capture the Ag NPs depending on the surface charge intensity of the nanoparticles [98]; then, their accumulation in the cells likely damages the mitochondrial membrane due to oxidative stress, thereby damaging the DNA [99,100,101]. Moreover, the induction of cell apoptosis is considered [102]. Thus, knowledge on the toxicity of Ag NPs to several human cells has continued to increase. However, related research mainly focuses on in vitro and animal experiments and is rarely reported. For example, in vivo acute/subacute toxicity data showed no changes in mice health status after intraperitoneal administration. Histological observations of internal organs and the biochemical parameters analyzed together with the other biological observations showed a low toxicity level with no major differences related to control, albeit at skin level a reduced number of mast cells was detected [92]. Hence, further studies on safety to humans are expected in the future [103,104].

## 6. Applications for Healthcare Workers (HCWs)

“Emerging” infectious diseases (EIDs) can be defined as infections that have newly appeared in a population or have existed but are rapidly increasing in incidence or geographic range [105]. Among recent examples are Ebola Virus Disease (EVD), Middle East Respiratory Syndrome coronavirus (MERS-CoV), Severe Acute Respiratory Syndrome (SARS), infection with MRSA, and Cholera. The HCWs involved during medical treatment of EID patients have a fatal risk of contact infection. The EID outbreak in west Africa had a devastating effect on HCWs. Of the nearly 17,000 cases of EVD in Guinea, Liberia, and Sierra Leone, at least 600 were among HCWs, and more than half of them died [106]. MERS-CoV infection continues to have a high fatality rate, and a large proportion of patients are HCWs (26%) [107].

**Figure 3:**
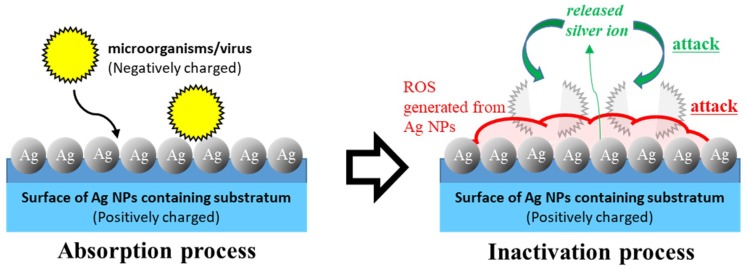
The mechanism for microbicidal and antivirus activities of the Ag NP chitin nanofiber sheet (CNFS). To prevent contact infection of healthcare workers (HCWs), an Ag NP chitin nanofiber sheet (CNFS) was developed, showing strong microbicidal activity against microorganisms/viruses via reactive oxygen species (ROS) and silver ions on the surface of substratum.

As described above, Ag NPs have a strong microbicidal activity with a broad spectrum. Furthermore, the mechanism that has been proposed is that Ag NPs yield ROS, leading to oxidative stress [100,101] in addition to the generation of free silver ions [48]. Therefore, Ag NPs will provide useful materials to protect HCWs from the risk of contact infection. To prevent contact infection, HCWs usually wear protective clothing. Pathogenic microbes, which are mainly generated by patients, stay alive on the surfaces of protective clothing. It was necessary to develop an evidence-based protective clothing for HCWs [108]. Especially, there is a risk of infection by incorrect contact when removing the clothing. To overcome that problem, we carried out research with the aim of developing a new microbicidal/antiviral material, using Ag NPs absorbed on a chitin sheet with a nanoscale fiber-like surface structure (Figure 3) [48,109].

The chitin nanofiber sheet (CNFS) used in our study has a nanoscale fiber-like surface structure, with corresponding increases in the available surface area for adsorption of Ag NPs. In addition, the advantages, in terms of biochemical activities, of chitin/chitosan-based materials include anti-infectious activity, the stimulation of angiogenesis/wound repair, and the stabilization/activation of growth factors [110,111,112,113,114]. Recent studies show that the application of CNFS to skin improved the epithelial granular layer and increased granular density, suggesting the potential use of CNFS as a component of skin-protective formulations [31].

**Figure 4:**
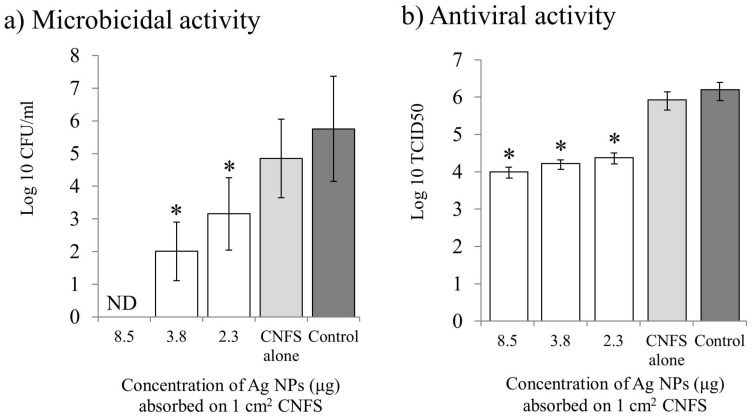
The microbicidal/antiviral activity of materials using Ag NPs/CNFS with various concentration of Ag NPs [48]. The activities of various concentrations of Ag NPs on CNFS against *E. coli* (**a**) and H1N1 Influenza A (**b**). Data are mean value ± standard deviation (*n* = 6); the asterisk indicates a statistically significant difference (*p* < 0.01) using two-sample t-test vs. control. ND means non-detection. The vertical axis is listed with a common logarithm. For example, with an Ag NP concentration of 8.5 μg/cm^2^ in the CNFS in (**b**), there was a reduction of greater than 2 log10 (100-fold) corresponding to a reduction of viral titers by approximately 99%.

**Figure 5:**
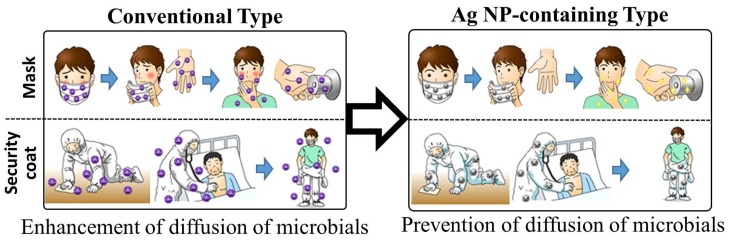
The application of the Ag NP/CNFS complex to protect HCWs. To prevent contact infection of HCWs, we have proposed medical consumables, such as infection-protective coats, masks, and gloves, immobilized Ag NPs.

We found that negatively charged Ag NPs are efficiently absorbed onto positively charged chitin/chitosan-based materials with a nanoscale fiber-like surface structure (<200 μm), such as CNFS, which also act as stabilizers of the Ag NPs [7,8,109]. Moreover, Ag NPs were able to bind directly to cotton, paper, and cloths with nanoscale fiber-like surface structures (data not published). Materials with immobilized Ag NPs have enhanced microbicidal activities against microbial pathogens and viruses. For example, we confirmed strong microbicidal activities of Ag NPs absorbed on CNFS against bacteria (*E. coli*) and viruses (H1N1 influenza A virus) (Figure 4) [9,48,109]. Thus, the materials have great potential to be used in clothes, plastics, and papers, with various applications such as in doctor/nurse uniforms, security/protection coats, masks, gloves, and counter cloths (Figure 5) [48]. Although we successfully synthesized new microbicidal/antiviral materials using Ag NP technology, further in-depth research will be required to evaluate how long these materials can maintain their stability in terms of microbicidal properties and to evaluate their safety for the human body. The issues of the materials’ safety for the human body is especially important, because the potential effects on patients who come into contact with the materials, through their various applications used by HCWs, should also be considered.

## 7. Other Medical Applications

Some of the commercially available silver-based materials already available are shown in Table 3. The range of microbicidal properties of Ag NPs is wider than that of antibiotics, and the appearance of resistant bacteria such as MRSA is rare in Ag NP-based materials [115]. The combination of various materials with Ag NP-based materials has been studied [116,117,118,119]. The production of wound dressings and medical catheters coated with Ag NPs has been investigated. However, silver absorbed by the skin causes food poisoning symptoms [120]. Moreover, metal allergies can be triggered if Ag NPs stay in the body for a long time [121]; therefore, care must be taken to avoid their intake.

The skin is the outermost tissue covering the human body and is prone to various stimuli and injuries [122]. Depending on the severity of the physical or chemical injury, the wound may hurt for a long time or even be lethal [123]. When caring for a wound, it is important to prevent it from drying out and to take measures against bacterial infection [124]. Wound dressings are used to maintain a humid environment, and disinfection and antibiotic treatment are measures against infection. Because of the worldwide concern regarding resistant bacteria due to the frequent use of antibiotics, a significant number of studies have been conducted on the development of wound dressings containing materials with antibacterial activity [125]. Wound dressings with Ag NPs are a representative example, and many types are now known. Silver ions are released from the Ag NPs contained in the wound-covering material in order to destroy the bacteria at the wound site [126].

Various types of catheters are used in the medical field; for example, the central venous catheter (CVC) is used intravenously from the body surface, near the main vein in the region of the right atrium for treatment or nutritional supplementation [127,128,129]. The highest possible attention is thus required to accurately locate the blood vessel and avoid any risk of bacterial infection by the catheter [129]. There is also a risk of septicemia if bacterial infection is caused via a catheter. More than 80% of infectious cases have been caused by MRSA [130]. Catheters coated with Ag NP have been developed to protect patients from such infections [131,132,133]. Approaches for preventing infections caused by both Gram-negative and Gram-positive bacteria have been reported [128,134,135,136]. There has been a continuous improvement in catheters coated with Ag NPs, and the development of devices with bactericidal properties and suppressed toxicity to the organism has advanced [129,134,137]. Urinary catheters are also frequently used in hospitals. Usually, these are made of silicone or latex and indwelled in the bladder via the urethra when the patient cannot urinate, e.g., when anesthetized or when the urine volume must be strictly measured [138]. Due to the nature of urinary catheters, bacterial infections in the urinary tract may arise [139]. Gram-negative bacteria, such as *E. coli*, *Enterococcus faecalis*, and *S. epidermidis*, frequently cause such infections [140]. A urinary catheter coated with Ag NPs exhibited hydrophilic properties and prevented the accumulation of proteins and electrolytes, suppressing the formation of biofilms [115].

An example of a typical use of silver materials in modern medicine is the amalgams that have been used in dentistry since the 19th century [141]. Currently, silver is also used in dental prostheses and implants, such as artificial dental roots implanted in the jawbone [142]. Dental implants are often contaminated by a biofilm, resulting in severe inflammatory disorders [143]. To overcome this, various metal dental implants or dental implants coated with metals have been tested, but favorable results were only obtained when using silver [144].

## 8. Conclusion Remarks

Applied research on Ag NPs in the biomedical field has been actively conducted, of which only a portion has been introduced in this review. There are high expectations regarding nanoparticles’ use; however, the toxic effect of Ag NPs on living organisms and the related health problems are concerning when their concentration exceeds a certain level [22]. For example, high blood pressure may be caused by Ag NPs [145]. Eliminating silver accumulated in the body is difficult. However, when the silver is present as nanoparticles, the toxicity decreases because they can be eliminated through the urine and hair [146]. However, current knowledge on the toxicity of Ag NPs to humans is based on in vitro tests and animal experiments, and there is no strict consensus about their toxicity. Therefore, the safety of Ag NPs when used for the human body needs further investigation. The microbicidal spectrum of Ag NPs is wide, and there is little concern regarding the rise of resistant bacteria. Recent studies revealed their effect on viruses that were thought to be unaffected by nanoparticles. The application of Ag NPs in the medical field is fascinating, particularly for infectious diseases. Attempts have also been made to impart microbicidal properties to biocompatible medical devices, and Ag NPs are considered to be one of the materials that will contribute to the progress of medical science in the future. In addition, materials based on Ag NP technology are expected to contribute to research into protecting HCWs from various risks, such as contact infection during medical treatments on patients. We expect that Ag NP-based materials will be able to prevent the contact infection of HCWs, besides preventing patient infection.

## Figures and Tables

**Table 1:** Some green synthesis methods for synthesizing Ag NPs.

Material	Size (nm)	Note
Bacteria	28–122	*E. coli* [23]
	10–15	*Rhodococcus spp.* [24]
	44–143	*Bacillus thuringiensis* [25]
	38–85	*Ochrobactrum anhtropi* [26]
	8.1–91	*Pantoea ananatis* [27]
	41–68	*Bacillus brevis* [28]
	105	*Bacillus mojavensis* [29]
Fungi	1–20	*Aspergillus terreus* [30]
	8–50	*Pleurotus ostreatus* [31]
	25–50	*Bryophilous rhizoctoni* [32]
	10, 50	*Penicillium fellutanum* [33]
	7	*biomass derived from Aspergillus flavus* [34]
	14, 25	*Penicillium expansum* [35]
plant	9	*Jasminum nervosum* [36]
	10–40	*Artemisia princeps* [37]
	20	*Cassia auriculata* [38]
	34	*Eclipta prostrata* [39]
	20, 30	*Coffea arabica* [40]
	10–60	*Antigonon leptopus* [41]
	25–40	*Fraxinus excelsior* [42]

**Table 2:** Some chemical methods for synthesizing Ag NPs.

Method	Size (nm)	Note
Chemical reduction	<50	Hydrogen peroxide was used as reducing agent [12].
	7.6–13.11	Sodium borohydride was used as reducing agent [49].
	7, 29, 89	Gallic acid was used as reducing agent [50].
	<30	Sodium citrate was used as reducing agent [51].
	5, 7, 10, 15, 20, 30, 50, 63, 85, 100	Sodium borohydride and trisodium citrate were used as reducing agent [52].
	9, 11, 24, 30	Hydrazine hydrate and sodium citrate were used as reducing agent [53].
	∼5	Sodium borohydride and citrate were used as reducing agent [54].
Electrochemical synthesis	4.8	Dry oxygen-free solvents were used under an argon atmosphere. [55].
	1–18	The film, as a cathode, was ion exchanged to desired Ag contents in AgNO_3_ solutions and then reduced electrochemically [56].
	30, 46	A platinum was employed as cathode and anode [57].
Irradiation-assisted method	30–120	Dual-beam illumination system (546 nm/440 nm) was used [58].
	2–8	Ag NPs were synthesized with UV (266 nm) irradiation [59].
	50	Ag NPs were synthesized by a microwave irradiation (Cu-Kα; 0.154 nm at 40 kV) [60]
	3–30	Ag NPs containg hydrogels were prepared by radiation crosslinking and reduction, simultaneously [61].
Pyrolysis method	20–300	An argon gas was used under oxygen-free environment [62].
	3–150	All solutions were dispersed by oxygen environment [63].

**Table 3:** Some of the commercially available Ag NP-based materials for clinical use.

Type	Name of Product	Company	Note
Wound dressing material	Acticoat™	Smith & Nephew, Inc., London, UK.	Nanocrystalline silver is used as a dressing to manage wounds by providing broad-spectrum bactericidal activity against over 150 pathogens.
Wound dressing material	PolyMem Silver^®^	Ferris Mfg. Corp., Texas, USA.	Contains nanocrystalline silver particles, which act on bacteria within the dressing.
External Ventricular Drain Catheter	Silverline^®^ Ventricular Drainage Catheter	Spiegelberg GmbH & Co. KG., Hamburg, DEU.	The special silver additive reduces the possibility of microbial colonization of the product surface.
Drug Delivery Catheter	ON-Q SilverSoaker^TM^	Halyard Health, Inc., Georgia, USA.	The catheter has a silver nanoparticle coating which protects against the formation of infection-causing biofilm.
Endotracheal Tube	Agento^®^ I.C. silver-coated endotracheal tube	C.R. Bard Inc., New Jersey, USA.	With a hydrophilic polymer coating containing silver particles, it was proven to reduce microbiologically confirmed ventilator-associated pneumonia.

## Abbreviations

Ag NPs	silver nanoparticles
CAUTIs	catheter-associated urinary tract infections
CNFS	chitin sheet with nano-scale fiber-like surface structure
CVC	central venous catheter
EID	emerging infectious diseases
EPA	the Environmental Protection Agency of the United States
EVD	ebola virus disease
*E. coli*	*Escherichia coli*
EG	ethylene glycol
HaCaT	human keratinocyte cell line
HSV	herpes simplex virus
HIV	human immunodeficiency virus
MERS-CoV	middle east respiratory syndrome coronavirus
MPV	monkeypox virus
MRSA	methicillin-resistant Staphylococcus aureus
NAD	nicotinamide adenine dinucleotide
PFU	plaque-forming unit
PVP	polyvinylpyrrolidone
ROS	reactive oxygen species
SARS	severe acute respiratory syndrome
TEM	transmission electron microscopy
UV	ultraviolet
Vis	visible
